# An engineered xCas12i with high activity, high specificity, and broad PAM range

**DOI:** 10.1093/procel/pwac052

**Published:** 2022-11-15

**Authors:** Hainan Zhang, Xiangfeng Kong, Mingxing Xue, Jing Hu, Zikang Wang, Yinghui Wei, Haoqiang Wang, Jingxing Zhou, Weihong Zhang, Mengqiu Xu, Xiaowen Shen, Fengcai Yin, Zhiyuan Ai, Guangyan Huang, Junhui Xia, Xueqiong Song, Hengbin Li, Yuan Yuan, Jinhui Li, Na Zhong, Meiling Zhang, Yingsi Zhou, Hui Yang

**Affiliations:** HuiEdit Therapeutics Co., Ltd., Shanghai 200120, China; HuiEdit Therapeutics Co., Ltd., Shanghai 200120, China; HuiEdit Therapeutics Co., Ltd., Shanghai 200120, China; HuiEdit Therapeutics Co., Ltd., Shanghai 200120, China; HuiEdit Therapeutics Co., Ltd., Shanghai 200120, China; HuiEdit Therapeutics Co., Ltd., Shanghai 200120, China; HuiEdit Therapeutics Co., Ltd., Shanghai 200120, China; HuiEdit Therapeutics Co., Ltd., Shanghai 200120, China; HuiEdit Therapeutics Co., Ltd., Shanghai 200120, China; HuiEdit Therapeutics Co., Ltd., Shanghai 200120, China; HuiEdit Therapeutics Co., Ltd., Shanghai 200120, China; HuiEdit Therapeutics Co., Ltd., Shanghai 200120, China; HuiEdit Therapeutics Co., Ltd., Shanghai 200120, China; HuiEdit Therapeutics Co., Ltd., Shanghai 200120, China; HuiEdit Therapeutics Co., Ltd., Shanghai 200120, China; HuiEdit Therapeutics Co., Ltd., Shanghai 200120, China; HuiGene Therapeutics Co., Ltd., Shanghai 200120, China; HuiGene Therapeutics Co., Ltd., Shanghai 200120, China; HuiEdit Therapeutics Co., Ltd., Shanghai 200120, China; HuiEdit Therapeutics Co., Ltd., Shanghai 200120, China; Center for Reproductive Medicine, International Peace Maternity and Child Health Hospital, Innovative Research Team of High-level Local Universities in Shanghai, School of Medicine, Shanghai Jiao Tong University, Shanghai 200030, China; HuiEdit Therapeutics Co., Ltd., Shanghai 200120, China; HuiEdit Therapeutics Co., Ltd., Shanghai 200120, China; HuiGene Therapeutics Co., Ltd., Shanghai 200120, China

**Dear Editor,**

The clustered regularly interspaced short palindromic repeats-Cas (CRISPR-Cas) systems, including type II Cas9 and type V Cas12 systems, which serve in the adaptive immunity of prokaryotes against viruses, have been developed into genome-editing tools (Anzalone et al., 2020; Doudna, 2020). Compared with type II systems, the type V systems including V-A to V-K showed more functional diversity (Yan et al., 2019). Amongst them, Cas12i has a relatively smaller size (1,033–1,093 aa), compared to SpCas9 and Cas12a, and has a 5’-TTN protospacer adjacent motif (PAM) preference (Yan et al., 2019). Cas12i is characterized by the capability of autonomously processing precursor crRNA (pre-crRNA) to form short mature crRNA. Cas12i mediates the cleavage of dsDNA with a single RuvC domain, by preferentially nicking the non-target strand and then cutting the target strand (Yan et al., 2019). These intrinsic features of Cas12i enable multiplex high-fidelity genome editing. However, the natural variants of Cas12i (Cas12i1 and Cas12i2) showed low editing efficiency which limits their utility for therapeutic gene editing.

To address these limitations, we screened 10 natural Cas12i variants and found 1, xCas12i, with robust high activity in HEK293T cells. Engineering of xCas12i by arginine substitutions at the PAM-interacting (PI) REC and RuvC domains led to the production of a variant, high-fidelity Cas12Max (hfCas12Max), with significantly elevated editing activity and minimal off-target cleavage efficiency. In addition, we assessed the base editing efficiency of xCas12i-based base editor, and thus expanded the genome-editing toolbox. We further demonstrated that hfCas12Max could be an effective genome-editing tool *ex vivo* and *in vivo* via ribonucleoprotein (RNP) and lipid nanoliposomes (LNP), respectively, suggesting the excellent potential for therapeutic genome-editing applications.

In order to identify more Cas12i variants, we developed and employed a bioinformatics pipeline to annotate Cas12i proteins, CRISPR arrays, and predicted PAM preferences, and found 10 new CRISPR/Cas12i systems. To evaluate the activity of these Cas12i variants in mammalian cells, we designed a fluorescent reporter system that detected the increased enhanced green fluorescent protein (EGFP) signal intensity activated by Cas-mediated dsDNA cleavage or double-strand breaks (Fig. S1A). This system relied on the co-transfection of a plasmid coding for mCherry, a nuclear localization signal (NLS)-tagged Cas protein and its guide RNA (gRNA) or crRNA, and one coding BFP and activatable EGxxFP cassette, which is EGxx-target site-xxFP. EGFP activation was carried out by Cas-mediated DSB and single-strand annealing (SSA)-mediated repair. Using this system, we observed that a variant, xCas12i, with targeted crRNA induced significant activation of EGFP expression (Figs. 1A and S1B), and exhibited a higher editing frequency than LbCas12a or SpCas9 as determined by Fluorescence Activated Cell Sorter (FACS) analysis (Fig. 1A). The xCas12i variant was smaller in size compared to SpCas9 and LbCas12a (Fig. S2A). We explored the effects of spacer length on cleavage efficiency in xCas12i, and found that 17-22 nt was the optimal length for their activation (Fig. S2B). Considering the 5’-TTN PAM preference of Cas12i, we performed an NTTN PAM identification assay using the reporter system. We found that xCas12i showed a consistently high frequency of EGFP activation at target sites with 5’-NTTN PAM sequences, while LbCas12a had comparable activity at 5’-TTTN PAM, respectively (Fig. S2C).

**Figure 1.**
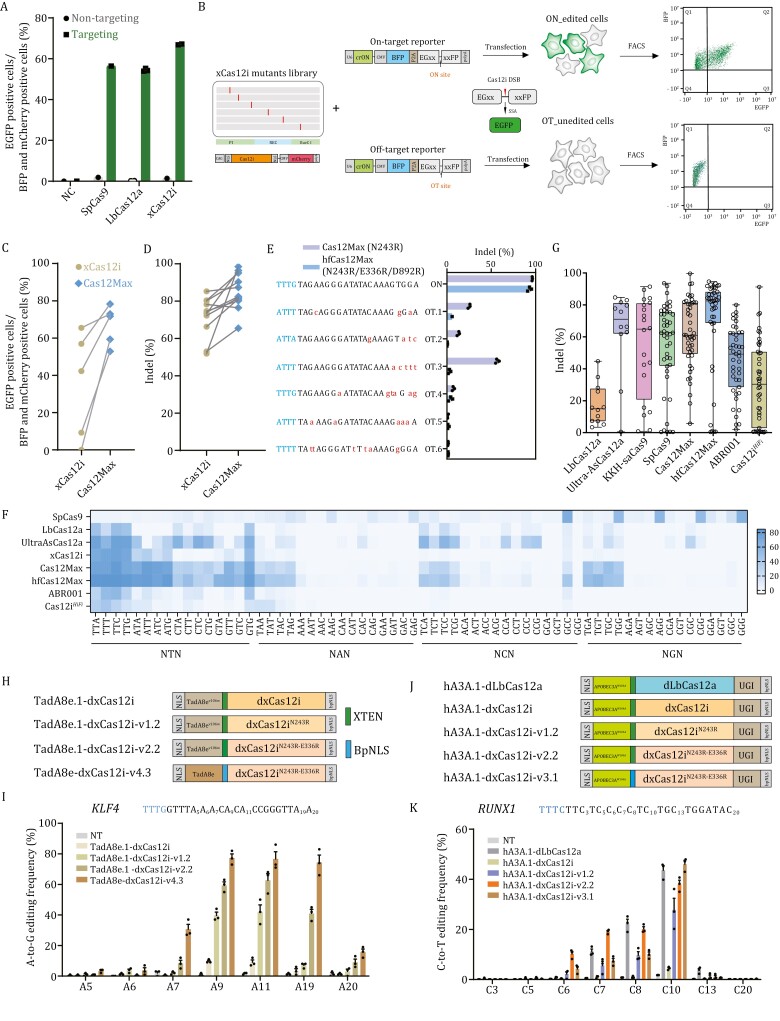
hfCas12Max, an engineered natural variant xCas12i, mediated high-efficient and -specificity genome editing, and dCas12i base editor exhibited high base editing activity in mammalian cells. (A) A natural variant xCas12i-mediated EGFP activation efficiency determined by flow cytometry. NC represents nonspecific control. (B) Schematics of protein engineering strategy for mutants with high efficiency and high fidelity using an activatable EGFP reporter screening system with on-targeted and off-targeted crRNA. (C and D) Cas12Max exhibited significantly increased cleavage activity than xCas12i at reporter plasmids (C) or various genomic (D) target sites. Each dot represents the mean indel frequency at one targeted site (*n* = 3). (E) NGS analysis showed that hfCas12Max retained comparable activity at TTR.2-ON targets and almost no at 6 OT sites, to Cas12Max. (F) Both Cas12Max and hfCas12Max exhibited a broader PAM recognition profile than other Cas proteins, including 5’-TN and 5’-TNN PAM. (G) Comparison of indel activity from Cas12Max, hfCas12Max, LbCas12a, Ultra AsCas12a, SpCas9 and KKH-saCas9 at TTR locus. hfCas12Max retained the comparable activity of Cas12Max, and higher gene-editing efficiency than other Cas proteins. Each dot represents one of three repeats of single target site. (H) Schematics of different versions of dxCas12i adenine base editors. (I) Comparison of A-to-G editing frequency at the KLF4 site from TadA8e.1-dxCas12i-v1.2, v2.2 and v4.3, v4.3 showed a high editing activity of 80%. TadA8e-dxCas12i-v4.3, named as ABE-dCas12Max. TadA8e.1 represents TadA8e V106W. (J) Schematics of different versions of dxCas12i cytosine base editors. (K) Comparison of C-to-T editing frequency at the DYRK1A site from hA3A.1-dxCas12i, -v1.2 v2.2 and v3.1, v3.1 showed a high editing activity of 50%. hA3A.1-dxCas12i-v3.1, named as CBE-dCas12Max. hA3A.1 represents human APOBEC3A W104A.

To further confirm the dsDNA cleavage activity of xCas12i in mammalian cells, we transfected an all-in-one plasmid encoding NLS tagged xCas12i with crRNAs targeting 37 sites from *TTR* (Gillmore et al., 2021), *PCSK9* in HEK293T or *Ttr* in N2a cells. The editing efficiency, that is, indel (insertion and deletion) formation at these loci was measured 48 h after transfection using FACS and targeted deep sequencing (Fig. S2D). We found that xCas12i mediated a high frequency, up to 90%, of indel formation at most sites from *Ttr*, *TTR*, and *PCSK9*, with a mean indel formation rate of over 50% (Fig. S2E, S2F, and Table S2). These data indicate that xCas12i exhibits a robust genome-editing efficiency in mammalian cells, suggesting it has excellent potential for therapeutic genome-editing applications.

To enhance its activity and expand its scope of PAM site recognition, we sought to engineer xCas12i protein via mutagenesis and screen for variants with higher efficiency and broader PAM using a reporter system, similar to what is described above. Substitution of an amino acid in the DNA-binding pocket with positively charged arginine (R) was shown to enhance the activity of the type V system (Kleinstiver et al., 2019). Combined with predictive structural analysis of xCas12i, we performed an arginine scanning mutagenesis approach in the PI, REC-I, and RuvC-II domains, generating a library of over 500 mutants (Figs. 1B and S3A). We then individually transfected these mutant variants with an activatable EGFP reporter system in HEK293T cells and analyzed them by FACS (Fig. 1B). Based on the fluorescence intensity of cells with activated EGFP, over 100 mutants showed an increased frequency of activated cells relative to wild-type (WT) xCas12i, and one mutant, named as Cas12Max, containing N243R showed a 3.4-fold improvement (Fig. S3A). We then performed saturation mutagenesis of N243, and found that the mutation to R showed the highest activity (Fig. S4A). We next targeted *DMD* or *Ttr* sites using the fluorescent reporter system, and found that Cas12Max displayed a markedly increased frequency of EGFP activation, relative to WT xCas12i (Figs. 1C, S4B, and S4C; Table S2). To further test the efficacy of Cas12Max in targeting genomic loci, we designed a total of eight gRNAs to target sites *TTR* and *PCSK9* in HEK293T cells and three more targeting *Ttr* in N2a cells. Consistent with our previous result, Cas12Max exhibited a significantly increased frequency of indels compared to WT xCas12i (Fig. 1D and Table S2).

To examine the specificity of Cas12Max, we transfected a construct designed to express it with crRNA targeting *TTR* (Gillmore et al., 2021), and performed indel frequency analysis of on- and off-target (OT) sites predicted by Cas-OFFinder (Bae et al., 2014). Using reporter system or targeted deep sequence analysis, we found that Cas12Max efficiently edited target sites and resulted in significant indel formation at two of three predicted off-target sites (Fig. S5). To eliminate the off-target activity of Cas12Max, we screened these mutants with mutations in the REC and RuvC domains, which have undiminished on-target cleavage activity, for those with no off-target activity, using two activatable reporter systems, each containing one OT site (Fig. 1B). We found that four mutants (v4.1-V880R, v4.2-M923R, v4.3-D892R, and v4.4-G883R) maintained a high level of on-target editing activity and showed significantly reduced off-target EGFP activation (Fig. S6A). We further combined these four amino acid substitutions with N243R and/or E336R of Cas12Max and found that the variant v6.3 (N243R/E336R/D892R) showed the lowest off-target EGFP activation at OT.1 and OT.2 sites and high on-target at the ON.1 site (Fig. S6B and S6C). Targeted deep sequencing analysis of endogenous TTR.2 site and its off-target sites in HEK293T showed that v6.3 (N243R/E336R/D892R) significantly reduced off-target indel frequencies at six OT sites and retained on-target at ON site, compared to Cas12Max (Fig. 1E). In addition, relative to Cas12Max (v1.1), v6.3 (N243R/E336R/D892R) retained comparable or even higher on-target activity at DMD.1, DMD.2, and DMD.3 sites (Fig. S6D). Therefore, we named v6.3 as high-fidelity Cas12Max (hfCas12Max).

Additionally, to investigate hfCas12Max’s PAM preference, we performed a 5’-NNN PAM recognition assay by designing reporter plasmids with the same target sequence but different PAM. Besides showing a consistent or higher cleavage activity at sites with a 5’-TTN PAM, hfCas12Max and Cas12Max showed a similarly high cleavage activity for targets with TNN, ATN, GTN, and CTN PAM sites, compared with the commonly used Cas12 (Zetsche et al., 2015; Zhang et al., 2021) (LbCas12a, Ultra-AsCas12a) and recently reported improved Cas12i2 (Chen et al., 2022; McGaw et al., 2022) (ABR001, Cas12i2^*HiFi*^) (Fig. 1F). Taken together, these results demonstrate that hfCas12Max exhibits high-efficiency editing activity with highly flexible 5’-TN or 5’-TNN PAM recognition.

To comprehensively evaluate the performance of hfCas12Max in human cells, we designed a large number of target sites in the exons of *TTR* for various Cas nucleases. In total, editing activity was monitored 43 sites for hfCas12Max with TTN PAMs, 43 sites for ABR001 with TTN PAMs, 43 sites for Cas12i2^*HiFi*^ with TTN PAMs, 45 sites for SpCas9 with NGG PAMs, 12 sites for LbCas12a with TTTN PAMs, 12 sites for Ultra AsCas12a with TTTN PAMs, and 20 sites for KKH-saCas9 with NNNRRT PAMs. Indel analysis showed that hfCas12Max exhibited an average efficiency of 70%, higher activity than other Cas nucleases, and comparable activity with Cas12Max (Figs. 1G and S7; Table S3). To further evaluate the specificity of hfCas12Max in human cells, we determined indel frequencies of *P2RX5* and *NLRC4* on-target and their corresponding *in silico* predicted off-target sites. Targeted deep sequence analysis showed that hfCas12Max had a higher on-target editing efficiency and similarly almost no indel activity at potential off-target sites, compared to Ultra AsCas12a and LbCas12a (Fig. S8A and S8B). To sufficient detect off-target of hfCas12Max and to compare to other Cas proteins, we used PEM-seq (Yin et al., 2019) to quantify germline events (uncut or perfect rejoining) and editing events including indels and translocations events of TTR.2 libraries. We found that LbCas12a, Ultra-Cas12a, ABR001, and hfCas12Max showed similar on-target editing efficiencies and a similar percentage of off-target translocations, while Cas12i2^*HiFi*^ showed both low on-target and off-target editing efficiencies (Fig. S9). Overall, these results demonstrate that hfCas12Max has high efficiency and specificity and is superior to SpCas9 and other Cas12 nucleases.

We further explored the base editing of xCas12i by generating a nuclease-deactivation xCas12i (dxCas12i). This was done by first introducing single mutations (D650A, D700A, E875A, or D1049A) in the conserved active site of xCas12i based on alignment to Cas12i1 and Cas12i2 (Fig. S10A and S10B). Then, these variants were fused with TadA8e^V106W^ named as TadA8e.1, or human APOBEC3A^W104A^, named as hA3A.1, to form the dxCas12i base editors TadA8e.1-dxCas12i and hA3A.1-dxCas12i, respectively (Richter et al., 2020; Wang et al., 2020). The initial versions of TadA8e.1-dxCas12i and hA3A.1-dxCas12i showed low base editing activity with frequencies of 8% A-to-G and 2% C-to-T, respectively (Fig. 1L and 1K). To address this, we introduced single and combined mutations for high cleavage activity into the PI and Rec domains of dxCas12i, which resulted in significantly increased A-to-G editing activity (Fig. S11A). Among the improved variants, dxCas12i-TadA8e-v2.2 (N243R/E336R) achieved 50% activity at A9 and A11 sites of the *KLF4* locus, markedly higher than the 30% activity of dLbCas12a-TadA8e (Figs. 1L, S11B, and S11C). At target sites within *PCSK9* and *TTR*, TadA8e-dxCas12i-v2.2 showed a similarly increased efficiency to mediate A-to-G transitions, and higher than dLbCas12a-TadA8e at *PCSK9* site (Fig. S13A and S13B). To test whether the orientation of deaminase fusion affects the base editing efficiency, we constructed dxCas12i-ABE by fusing the TadA8e.1 to N- or C-terminus of dxCas12i, and found that TadA8e.1 at C-terminus of dxCas12i showed slightly higher activity than N-terminus (Fig. S12). We then further engineered the NLS, linker and TadA8e protein to produce TadA8e-dxCas12i-v4.3 which exhibited a nearly 80% A-to-G editing efficiency and >95% editing purity, while the editing activities of other dxCas12i-ABE versions were unchanged (Figs. 1H, 1I, S11D, S11E, and S13C). We named dxCas12i-TadA8e-v4.3 as dCas12Max-ABE. To further characterize the base editing activity of dCas12Max-ABE, we performed 21 sites with TTN PAM, 13 sites with ATN PAMs, and 13 sites with CTN PAMs. We found that dCas12Max-ABE exhibited significant A-to-G activity at sites with TTN PAM, but weak or hardly at ATN or CTN PAMs (Fig. S14). In addition, the substitution of dxCas12i-v1.2 (N243R), dxCas12i-v2.2 (N243R/E336R), or dxCas12i-v4.3 (N243R/E336R-bpNLS) showed consistently elevated C-to-T editing efficiency along with >95% editing purity, at C7 and C10 sites of RUNX1, DYRK1A, and SITE4 locus, even higher than hA3A.1-dLbCas12a at RUNX1 and DYRK1A (Figs. 1J, 1K and S15). These results together demonstrate that engineered dxCas12i-based editors exhibit high base editing activity in mammalian cells.

To explore the therapeutic potential application of hfCas12Max, we delivered hfCas12Max RNP targeting TRAC in CD3^+^ T cells (Zhang et al., 2021) (Fig. 2A). Beforehand, we tested hfCas12Max RNP targeting TTR and TRAC in HEK293 cells, and found that gene-editing efficiency was increased following the increasing dose of RNPs, with unaffected cellular viability and proliferation. We achieved 90% editing efficiency and >95% viability at 3.2 μmol/L dose (Fig. S16A–C) in HEK293 cells. Three guides were designed to target TRAC, and both sg.2 and sg.3 generated ~90% editing at both 1.6 and 3.2 μmol/L dose along with ~80% viability (Fig. 2B) in CD3^+^ T cells. Flow cytometric analysis showed that TRAC expression was detected to be reduced to a level of 2%–3% in CD3^+^ T cells post 5 days post-electroporation treated with RNPs targeting sg.2 or sg.3, compared to 96.6% with untreated cells (Fig. 2C).

**Figure 2.**
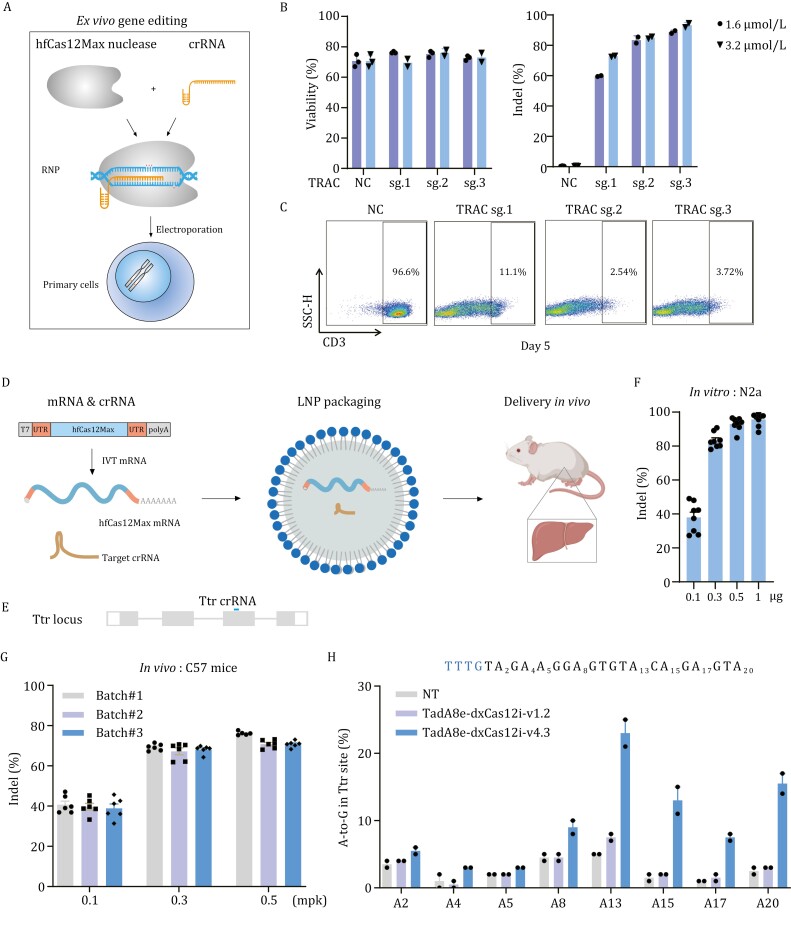
hfCas12Max mediates high-efficiency gene editing *ex vivo* and *in vivo*. (A) Schematics of hfCas12Max gene editing in primary human cells. (B) Viability and indel activity of human CD3^+^ T cells following delivery of hfCas12Max RNPs with three different *TRAC* targeted crRNAs at 1.6 and 3.2 μmol/L, respectively (*n* = 2 or 3). NC represents blank control, untreated with RNP. (C) Representative flow cytometric analysis of edited CD3^+^ T cell 5 days after RNP delivery. NC represents blank control, untreated with RNP. (D) Schematics of *in vivo* non-liposome delivery containing IVT-mRNA, LNP packaging process. (E) Editing efficiency of LNP packaging with hfCas12Max mRNA and targeted *Ttr* crRNA at increased concentrations in N2a cells (*n* = 8). (F) Schematics of *Ttr* locus. (G) Indel rates of LNP packaging with hfCas12Max mRNA and targeted *Ttr* crRNA at three dose (0.1, 0.3, and 0.5 mpk) in C57 mouse (*n* = 6). (H) The A to G editing percentage of LNP packaging with dCas12i-ABE mRNA and targeted *Ttr* crRNA at 3 mpk in C57 mouse (*n* = 2).

To assess the feasibility of hfCas12Max or base editor *in vivo* gene editing, we performed the LNP packaging mRNA and crRNA to deliver the liver in C57 mouse through tail intravenous injection (Fig. 2D). We targeted the exon 3 in the murine transthyretin (*Ttr*) gene by gene and base editing (Fig. 2E). Robust editing efficiencies were detected at four concentration and nearly 100% at 1 μg dose in N2a cells (Fig. 2F). Similarly, targeted deep sequence analysis indicated that the editing efficiencies of murine liver were approximately 70% at the dose of 0.3 and 0.5 milligrams per kilogram (mpk), equivalent to saturation (Fig. 2G). Further, through the LNP packaging delivery, TadA8e-dxCas12i-v4.3 achieved approximately 25% A-to-G efficiency of A13 in *Ttr* locus in murine liver at 3 mpk dose, while v1.2 only 8% (Fig. 2H). In addition, we injected hfCas12Max mRNA with two crRNAs targeting *Ttr* gene into murine zygotes, which were cultured to blastocyst stage for genotyping analysis (Fig. S17A). Targeted deep sequence analysis showed that most zygotes were edited and some up to 100% (Fig. S17B). These results indicate that hfCas12Max mediates robust *ex vivo* and *in vivo* gene editing, showing significant potential for disease modeling and therapies.

In this study, we demonstrate that the Type V-I Cas12i system enables versatile and efficient genome editing in mammalian cells. We found a natural Cas12i variant, xCas12i, that shows high editing efficiency at TTN-PAM sites. By semi-rational design and protein engineering of its PI, REC, RuvC domains, we obtained a high-efficiency, high-fidelity variant, hfCas12Max, which contains N243R, E336R, and D892R substitutions. In agreement with the hypothesis that introducing arginine at key sites could strengthen the binding between Cas and DNA, the introduction of N243R in the PI domain and E336R at REC domain significantly increased editing activity and expanded PAM recognition. Interestingly, D892R or G883R substitutions in the RuvC domain reduced off-target and retained on-target cleavage activity, whereas alanine substitutions (Bravo et al., 2022), which have been used to reduce off-target activity, did not (Fig. S6C). The D892R substituted hfCas12Max was obviously more sensitive to mismatch, which suggests that D892R or G883R improved sgRNA binding specificity. According to sequence alignment and predicted structure of xCas12i to Cas12i2, asparagine 892 is located on NUC domain, together with RuvC domain to form a cleft, in which crRNA:DNA heteroduplex was located. The variant with D892R did not alter the on-target but eliminated off-target activity, probably due to arginine substitution of asparagine affecting the binding of non-target crRNA. Our data suggest that a semi-rational engineering strategy with arginine substitutions based on the EGFP-activated reporter system could be used as a general approach to improve the activity of CRISPR editing tools.

Through engineering, our Cas12i system has achieved high editing activity, high specificity, and a broad PAM range, comparable to SpCas9, and better than other Cas12 systems. Given its smaller size, short crRNA guide, and self-processing features (Yan et al., 2019), the type V-I Cas12i system is suitable for *in vivo* multiplexed gene-editing applications, including AAV or LNP. Indeed, our data indicate type V-I Cas12i system mediates the robust *ex vivo* or *in vivo* genome-editing efficiencies via ribonucleoprotein (RNP) delivery and lipid nanoliposomes (LNP) delivery, respectively, demonstrating the great potential for therapeutic genome-editing applications.

In addition, we have confirmed that the type V-I Cas12i system can be used in base editing applications. For base editor, the dCas12i system shows high A-to-G editing at A9-A11 sites even A19 of KLF locus, and C-to-T editing at A7-A10 sites, which is similar to the dCas12a system but is distinct from the dCas9/nCas9 system. Comparable to dCas12a, dCas12i-BE exhibited higher base editing activity at KLF4, PCSK9, and DYRK1A loci (Figs. 1K, S13A, and S15A), suggesting it may have more potential as a base editor. This suggests that the dCas12i system is useful for broad genome engineering applications, including epigenome editing, genome activation, and chromatin imaging (Anzalone et al., 2020; Bock et al., 2022).

In summary, the Cas12i system described here, which has robust editing activity and high specificity, is a versatile platform for genome editing or base editing in mammalian cells and could be useful in the future for *in vivo* or *ex vivo* therapeutic applications.

## Supplementary data

The online version contains supplementary material available at https://doi.org/10.1093/procel/pwac052.

pwac052_suppl_Supplementary_Material

pwac052_suppl_Supplementary_Tables

## Footnotes

This work was supported by HUIGENE Therapeutics Inc and HUIEDIT Therapeutics Inc. We kindly thank Yuan Yuan, IP & Legal Director from HUIGENE Therapeutics Inc., for the helpful discussion.

H.Z., Y.Z. and H.Y. jointly conceived and designed the project. H.Z., H.W., J.Z., W.Z., M.X., and X.K. performed experiments with plasmid construction, cell culture and FACS, and data analysis. M.X., Z.W., F.Y., Z.A., G.H., and J.X. performed experiments with mRNA IVT, LNP packaging, and mice studies. H.J. and X.S. performed T-cells-related experiments via RNP electroporation. Y.W. and H.L. performed mice embryo-related experiments. X.S., Y.Y., J.L., N.Z., and M.Z. assisted with experiments. H.Z., Y.Z., and H.Y. co-supervised the whole project. H.Z., Y.Z., and H.Y. wrote and revised the manuscript.

The authors disclose a patent application relating to aspects of this work. Y.Z. and H.Y. are the founders of HUIEDIT Therapeutics Inc., and H.Y. is also the founder of HUIGENE Therapeutics Inc.

The authors have no conflicts of interest to declare that are relevant to the content of this article.

Whole genome sequencing data were deposited in Sequence Read Archive (SRA): PRJNA894938. Additional raw data for the figures in this manuscript can be obtained from the corresponding authors upon request.
