# Supplementary material for: An engineered xCas12i with high activity, high specificity, and broad PAM range
Source: Protein Cell. 2022 Nov 15;14(7):540–5. doi: 10.1093/procel/pwac052 (PMC10305737; doi:10.1093/procel/pwac052)
Supplement: pwac052_suppl_Supplementary_Material [file pwac052_suppl_supplementary_material.pdf]

Material and methods

Plasmid vector construction.

Human codon-optimized *Cas12i*, *TadA8e* and human *APOBEC3A* genes were synthesized by the GenScript Co., Ltd., and cloned to generate pCAG_NLS-Cas12i-NLS_pA_pU6_BpiI_pCMV_mCherry_pA by Gibson Assembly. crRNA oligos were synthesized by HuaGene Co., Ltd., annealed and ligated into *Bpi*I site to produce the pCAG_NLS-Cas12i-NLS_pA_pU6_crRNA_pCMV_mCherry_pA.

Cell culture, transfection and flow cytometry analysis.

The mammalian cell lines used in this study were HEK293T and N2A. Cells were cultured in Dulbecco's modified Eagle's medium (DMEM) supplemented with 10% FBS, penicillin/streptomycin and GlutMAX. Transfections were performed using Polyetherimide (PEI). For variant screening, HEK293T cells were cultured in 24-well plates, and after 12 hours 2 μg of the plasmids (1 μg Cas12i of a mutant plasmid and 1 μg of the reporter plasmid) were transfected into these cells with 4 μL PEI. 48 hours after transfection, mCherry and EGFP fluorescence were analyzed using a Beckman CytoFlex flow-cytometer. For assay of mutations in target sites of endogenous genes, 1 μg plasmid with Cas12i targeting crRNA was transfected into HEK293T or N2A cells, which were then sorted using a BD FACS Aria III, BD LSRFortessa X-20 flow cytometer, 48 hours after transfection.

Detection of gene editing frequency.

Six thousand sorted cells were lysed in 20 μl of lysis buffer (Vazyme). Targeted sequence primers were synthesized and used in nested PCR amplification by Phanta Max Super-Fidelity DNA Polymerase (Vazyme). Targeted deep sequence analysis was used to determine indel frequencies. A-to-G or C-to-T editing frequencies were calculated by targeted deep sequence analysis or Sanger sequencing and EditR. A-to-G editing purity were calculated as A-to-G editing efficiency/ (A-to-T editing efficiency + A-to-C editing

efficiency + A-to-G editing efficiency). C-to-T editing purity were calculated as C-to-T editing efficiency/ (C-to-A editing efficiency + C-to-G editing efficiency + C-to-T editing efficiency).

PEM-seq.

PEM-seq in HEK293 cells was performed as previously described[23]. Briefly, all-in-one plasmids containing LbCas12a, Ultra-AsCas12a, hfCas12Max, ABR001 or Cas12i2HiFi with targeting TTR.2 crRNA were transfected into HEK293 cells by PEI respectively, and after 48 hrs, positive cells were harvested for DNA extraction. The 20 μg genomic DNA was fragmented with a peak length of 300-700 bp by Covaris sonication. DNA fragments was tagged with biotin by a one-round biotinylated primer extension at 5'-end, and then primer removal by AMPure XP beads and purified by streptavidin beads. The single-stranded DNA on streptavidin beads is ligased with a bridge adapter containing 14-bp RMB, and PCR product was performed nested PCR for enriching DNA fragment containing the bait DSB and tagged with illumine adapter sequences. The prepared sequencing library was sequencing on an Hi-seq 2500, with a 2 x 150 bp.

RNP delivery and *ex vivo* editing.

RNP was complexed by mixing purified hfCas12Max proteins with chemically synthesized RNA oligonucleotides (Genscript) at a 1:2 molar ratio in 1X PBS. RNP was incubated at room temperature for >15 min prior to electroporation with Lonza® 4D-Nucleofector™. 0.2 × 10^6 cells were resuspended in 20 μL of Lonza buffer and mixed with 5 μL RNP with different concentrations electroporated according to Lonza specifications. HEK293 or CD3+ T cells were harvested 72 hrs post-electroporation for targeted deep sequence analysis.

LNP delivery and *in vivo* editing.

LNPs were formulated with ALC0315, cholesterol, DMG-PEG2k, DSPC in 100% ethanol, carrying *in vitro* transcription (IVT) mRNA and chemically synthesized RNA oligonucleotides (Genscript) with a 1:1 weight ratio. LNPs were formed according to the

manufacturer's protocol, by microfluidic mixing the lipid with RNA solutions using a Precision Nano-systems NanoAssemblr Benchtop Instrument. LNPs diluted in PBS were transfected into N2a cells at 0.1, 0.3, 0.5, 1 μg RNA, or delivered into C57 mouse with different dose by through tail intravenous injection. Cells were harvested 48 hrs post-transfection for lysis and targeted deep sequence analysis. For in vivo editing, liver tissue was collected from the left or median lateral lobe of each mouse 7 days post-injection for DNA extraction and targeted deep sequence analysis.

Zygote Injection and Embryo Culturing.

Super ovulated female C57 mice (7-8 weeks old) by injecting 5 IU of pregnant mare serum gonadotropin (PMSG), followed by 5 IU of human chorionic gonadotropin (hCG) 48 hrs later were mated to B6D2F1 males, and fertilized embryos were collected from oviducts 20 hrs post hCG injection. For zygote injection, hfCas12Max mRNA (100 ng/μL) and sgRNA (100 ng/μL) were mixed and injected into the cytoplasm of fertilized eggs in a droplet of HEPES-CZB medium containing 5 mg/ml cytochalasin B (CB) using a FemtoJet microinjector (Eppendorf) with constant flow settings. The injected zygotes were cultured in KSOM medium with amino acids at 37℃ under 5% CO_2 in air to blastocysts and harvested for targeted deep sequence analysis.

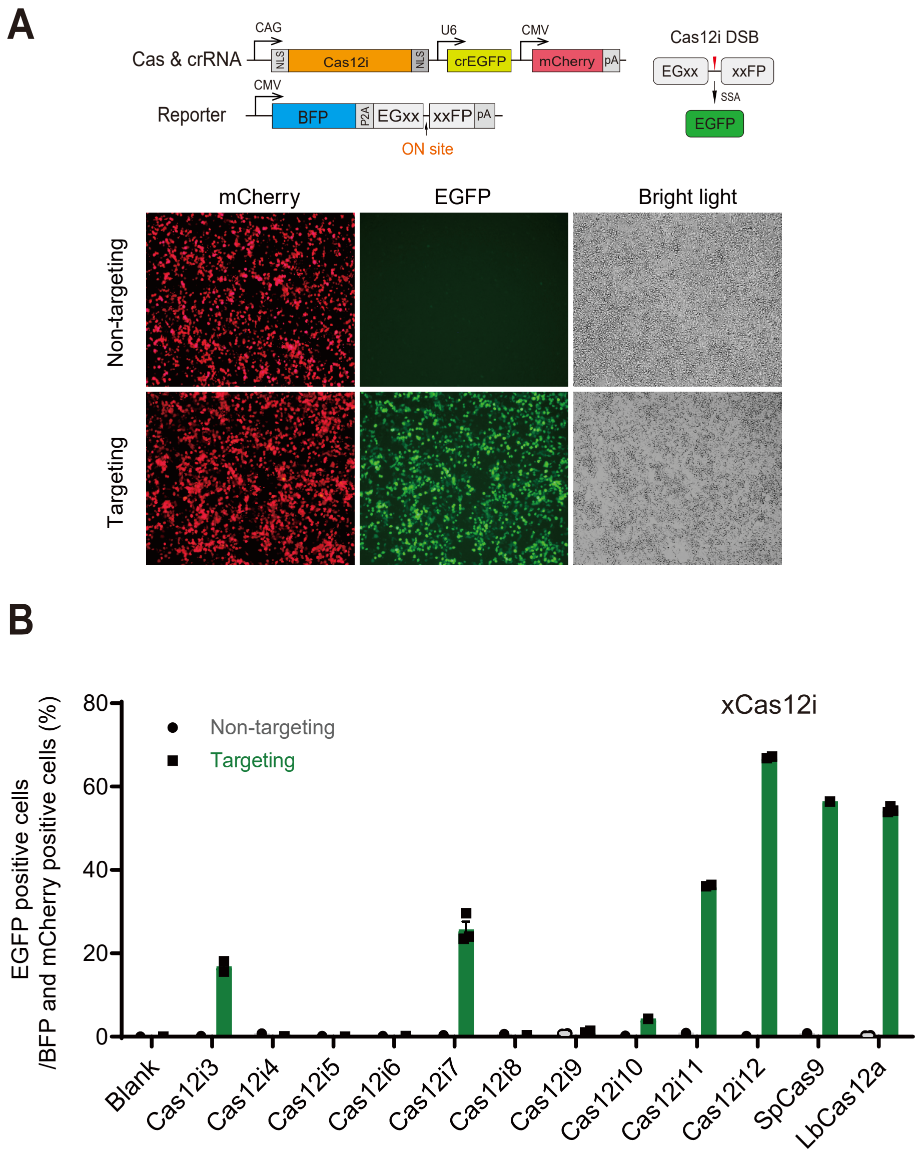

Supplementary Figure 1. Screen for functional Cas12i in HEK293T cells. A, Transfection of plasmids coding Cas12i and crRNA mediate EGFP activation. **B,** Five of ten natural Cas12i nuclease mediated EGFP-activated efficiency in HEK293T cells.

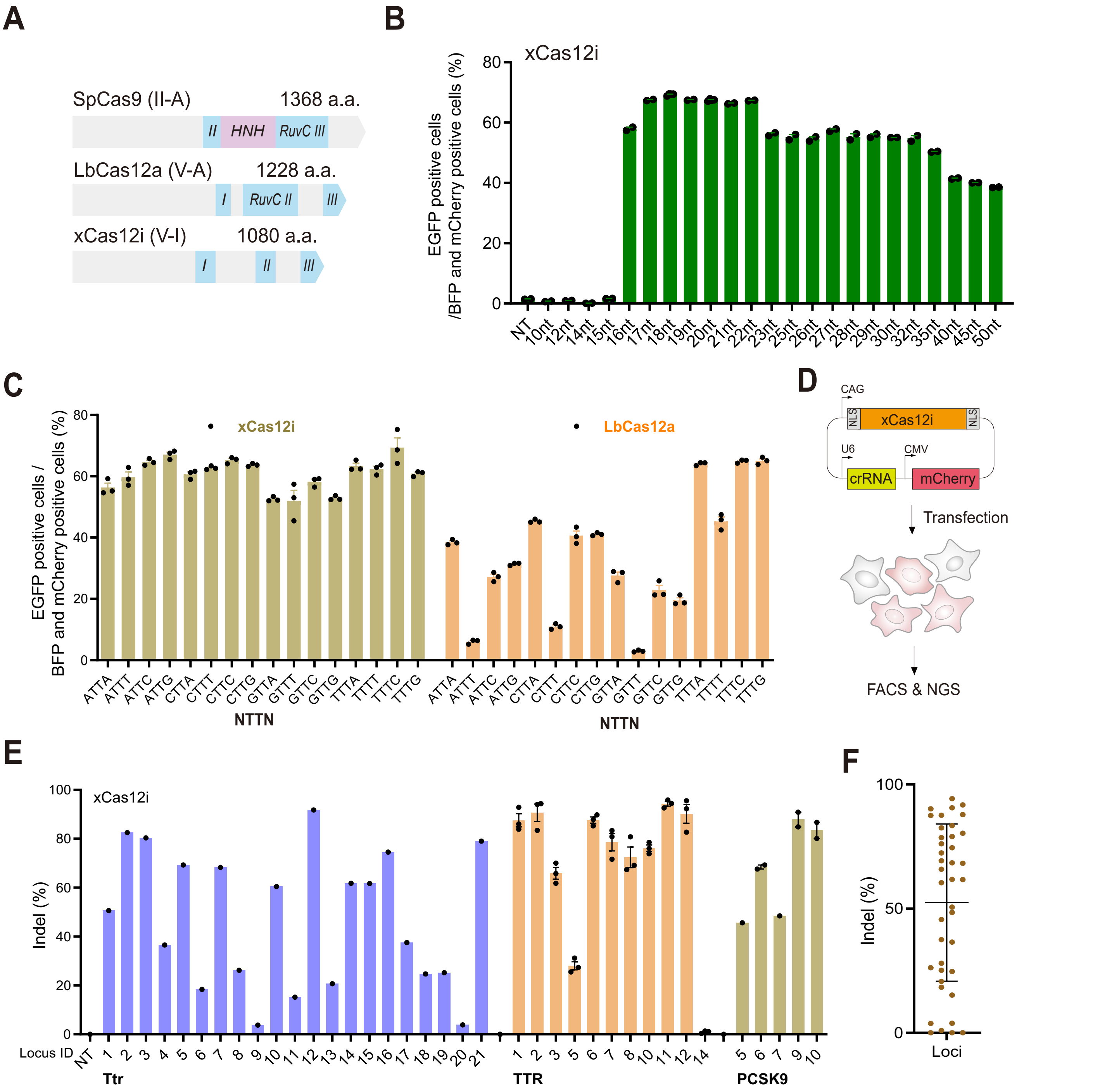

Supplementary Figure 2. Identification and characterization of type V-I systems. A, Nuclease domain organization of SpCas9, LbCas12a, and xCas12i. **B,** Optimal spacer length for xCas12i. **C**, PAM scope comparison of LbCas12a, and xCas12i. xCas12i exhibited a higher editing efficiency at 5’-TTN PAM than Cas12a. **D**, Flow diagram for detection of genome editing efficiency by transfection of an all-in-one plasmid containing xCas12i and targeted gRNA into HEK293T cells, followed by FACS and NGS analysis. **E-F**, xCas12i mediated robust genome editing (up to 90%) at the Ttr locus in N2a cells and TTR and PCSK9 in HEK293T cells.

A

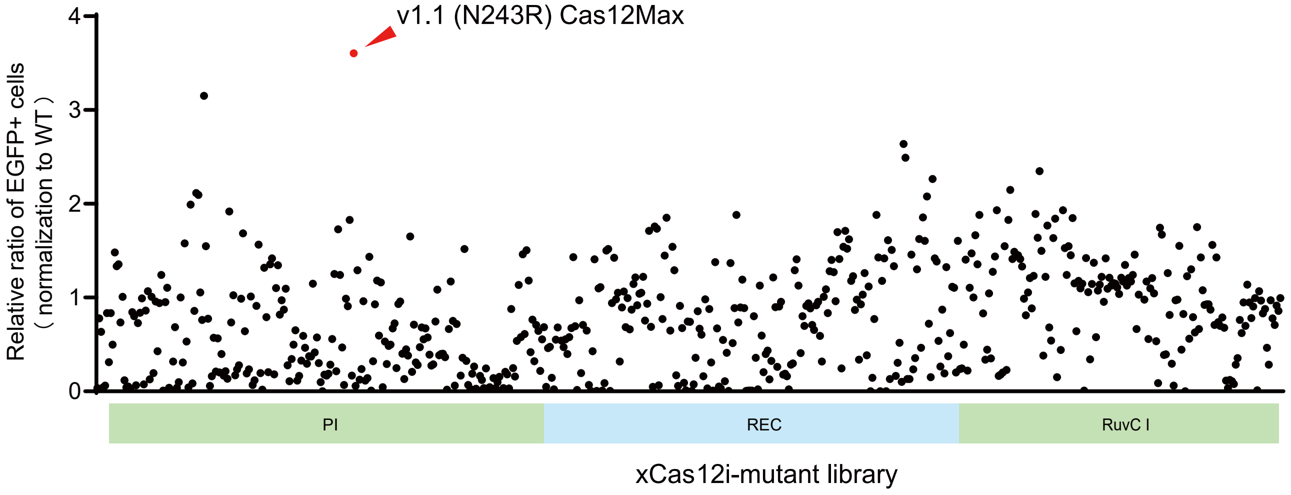

Supplementary Figure 3. Screen for engineered xCas12i mutants with high-efficiency editing activity. A, The relative editing frequencies of over 500 rationally engineered xCas12i mutants. v1.1 represents xCas12i with N243R, named as Cas12Max.

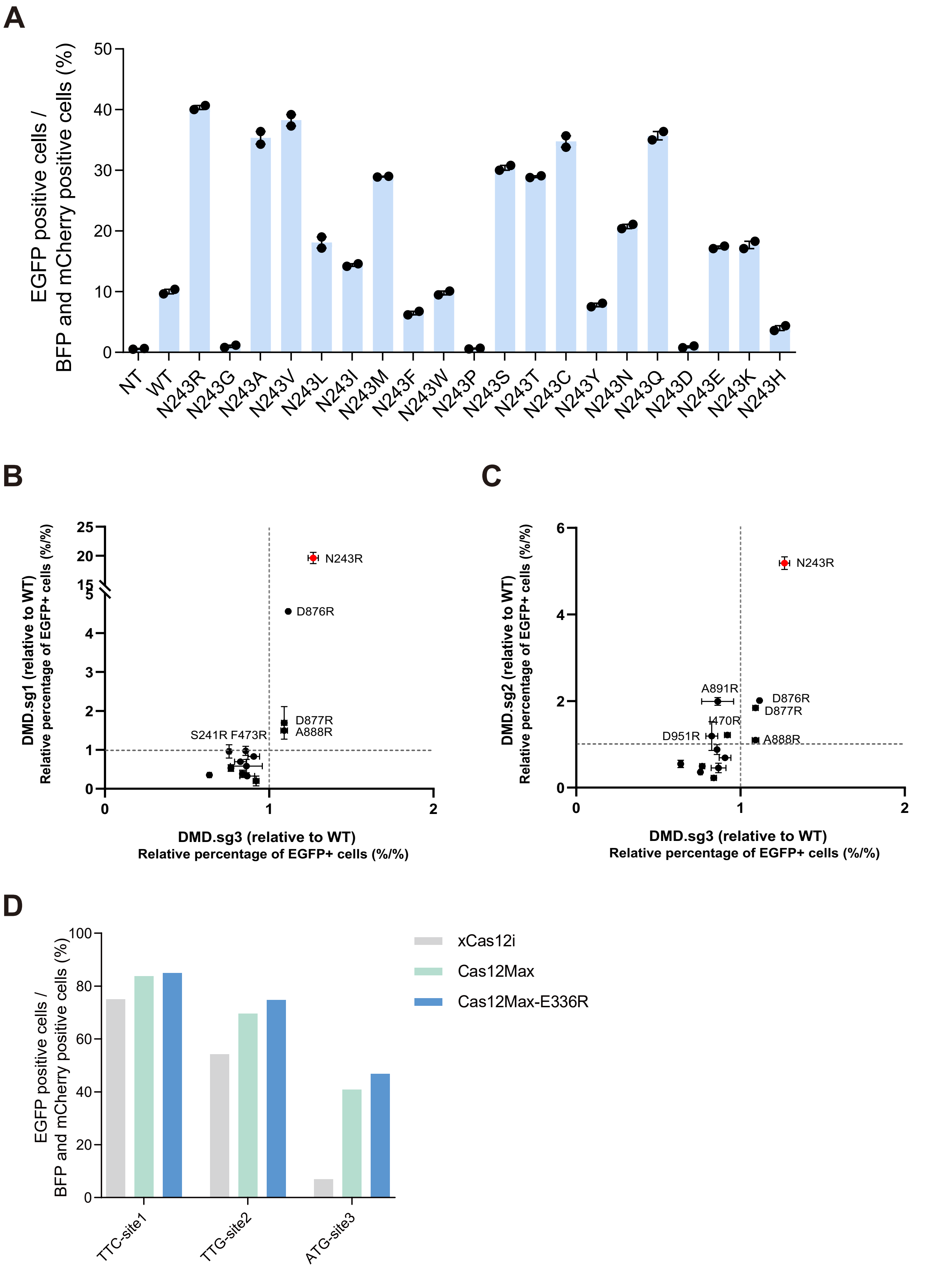

Supplementary Figure 4. Other mutants mediated high-efficiency editing. A, Of the saturated mutants of N243, N243R increased the EGFP-activated fluorescent most. **B-C,** xCas12i mutant with N243R increased 1.2, 5, 20-fold activity at DMD.1, DMD.2 and DMD.3 locus. **D,** Both Cas12Max (xCas12i-N243R) and Cas12Max-E336R elevated EGFP-activated fluorescent at different PAM recognition sites.

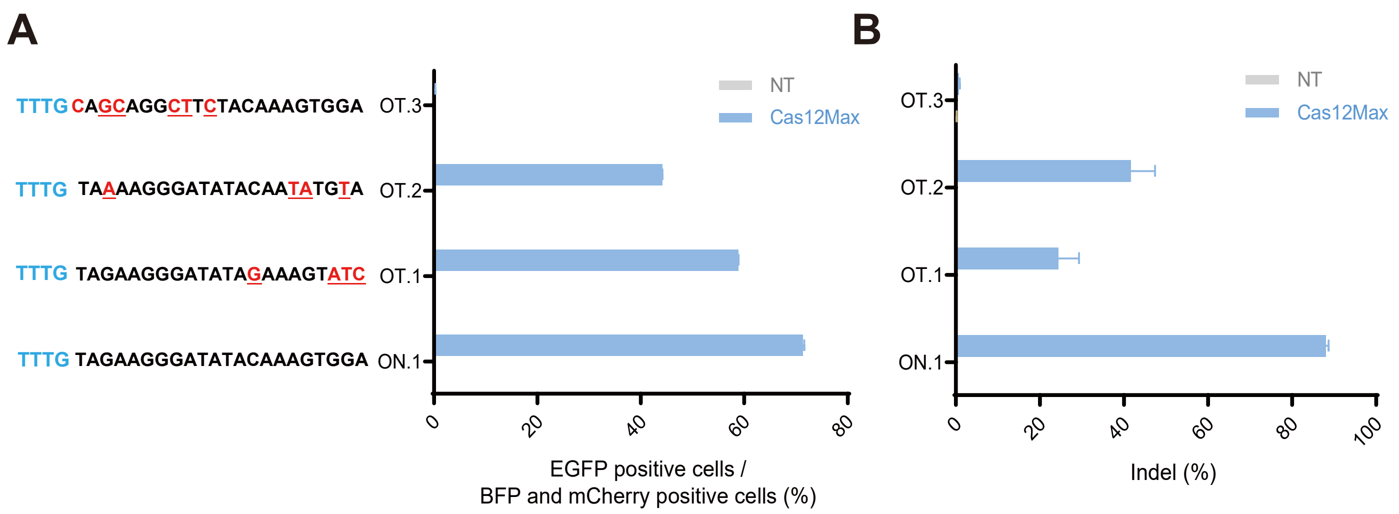

Supplementary Figure 5. Cas12Max induced off-target editing efficiency at sites with mismatches using the reporter system (A) and targeted deep sequence (B).

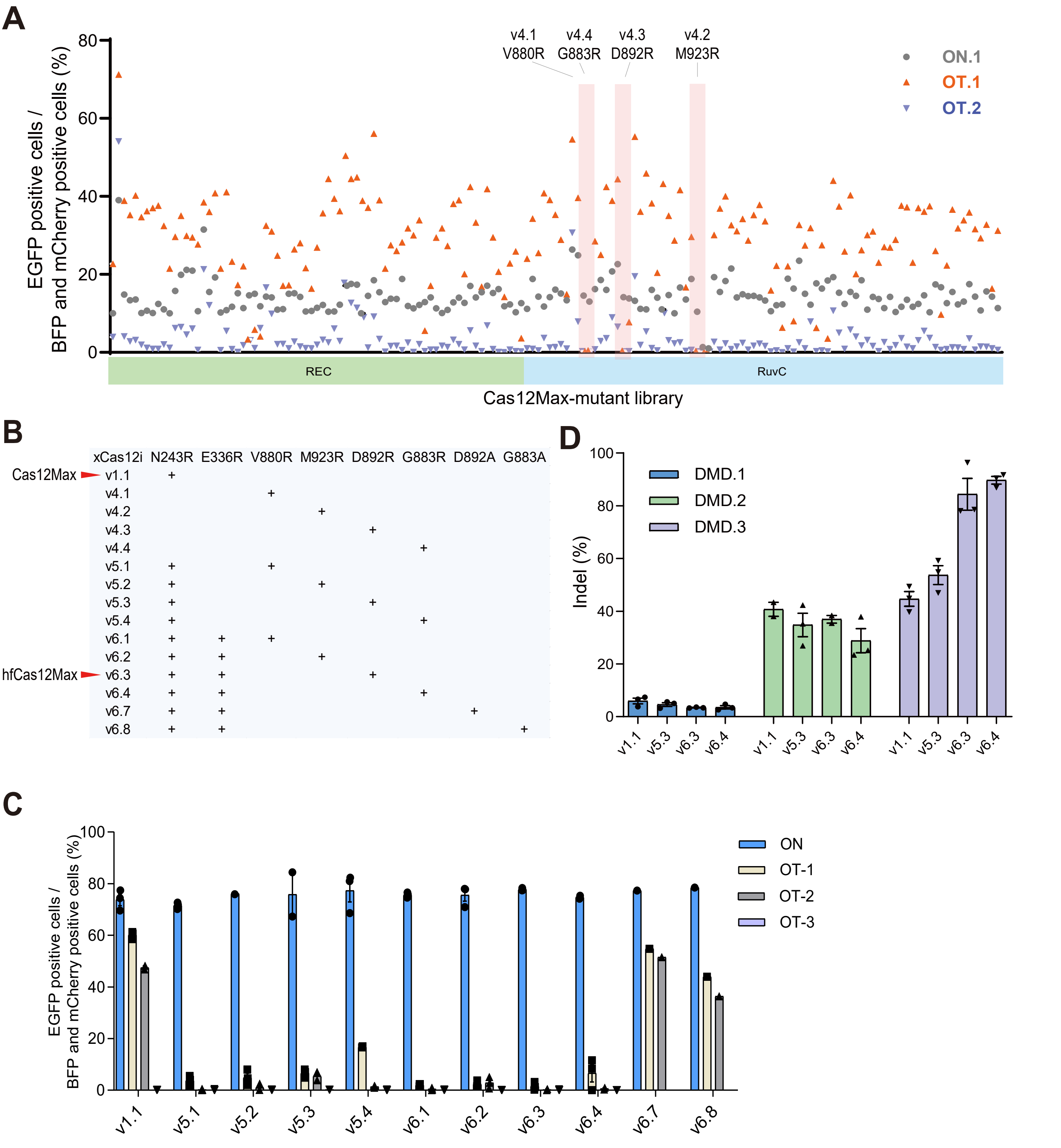

xCas12i	N243R	E336R	V880R	M923R	D892R	G883R	D892A	G883A
v1.1	+							
v4.1			+					
v4.2				+				
v4.3					+			
v4.4						+		
v5.1	+		+					
v5.2	+			+				
v5.3	+				+			
v5.4	+					+		
v6.1	+	+	+					
v6.2	+	+		+				
v6.3	+	+			+			
v6.4	+	+				+		
v6.7	+	+					+	
v6.8	+	+						+

Supplementary Figure 6. hfCas12Max mediates high-efficiency and -specificity editing. A, Rational protein engineering screen of over 200 mutants for highly-fidelity Cas12Max.Four mutants show significantly decreased activity at both OT (off-target) sites and retains at ON.1 (on-target) site. **B**, Different versions of xCas12i mutants. **C**, v6.3 reduced off-target at OT.1, OT.2 and OT.3 sites and retained indel activity at TTR-ON targets, compared to v1.1-Cas12Max. **D**, v6.3 exhibited comparable indel activity at DMD.1, DMD.2, and higher at DMD.3 locus, than v1.1-Cas12Max. v1.1, named as Cas12Max. v6.3, named as hfCas12Max.

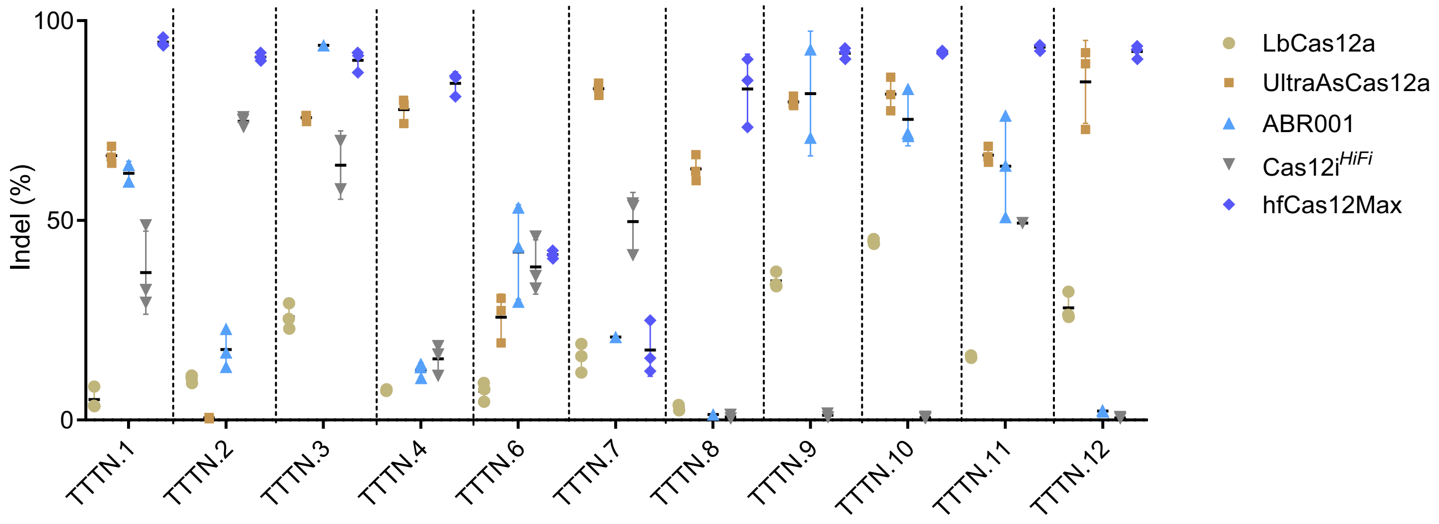

Supplementary Figure 7. Comparison of the gene-editing efficiency of hfCas12Max with LbCas12a, Ultra AsCas12a, ABR001 and Cas12i^{HiFi} at TTR locus.

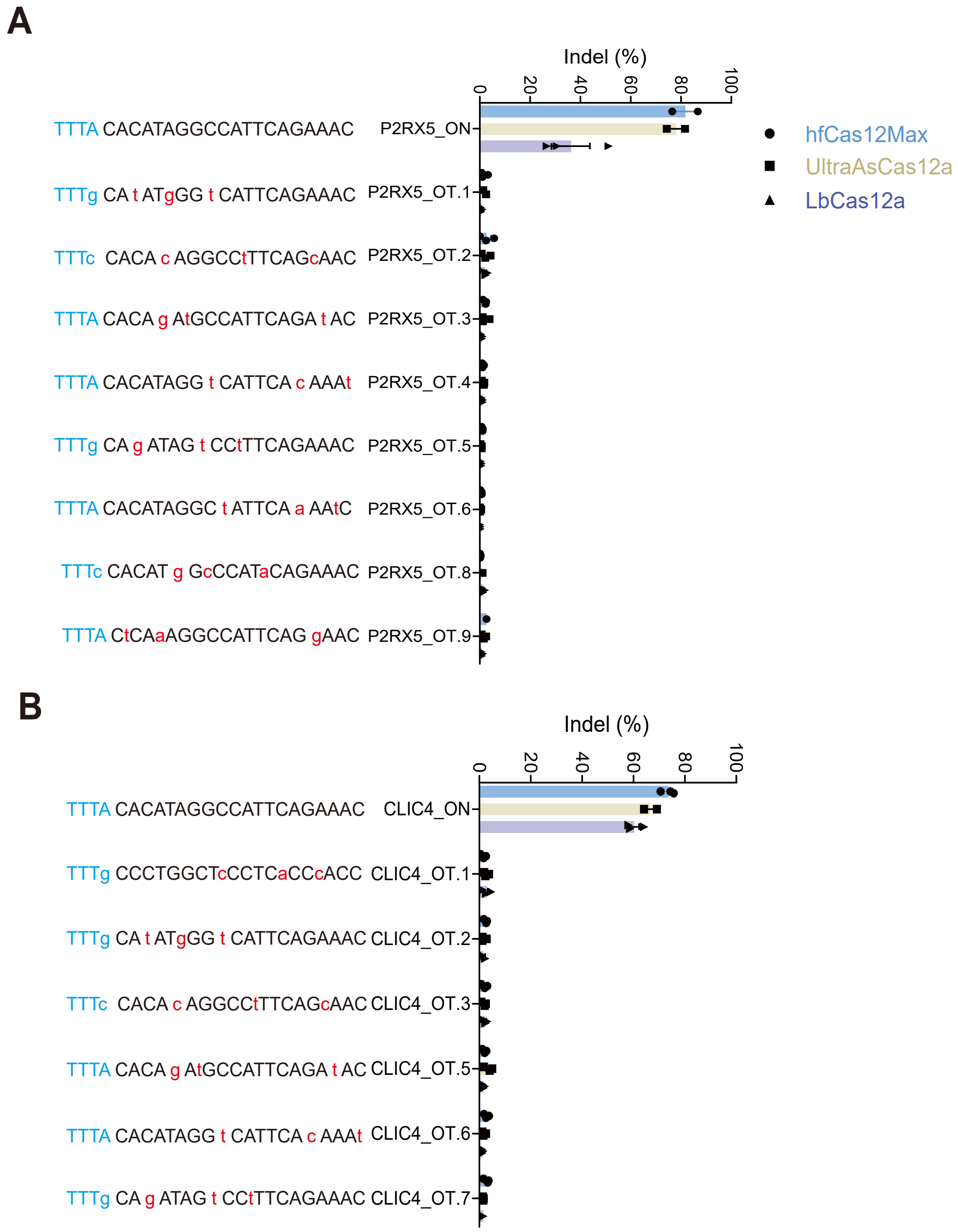

Supplementary Figure 8. hfCas12Max mediated the high-efficient and -specific editing. A-B, Off-target efficiency of hfCas12Max, LbCas12a, and UltraAsCas12a at *in-silico* predicted off-target sites, determined by targeted deep sequencing. Sequences of on-target and predicted off-target sites are shown, PAM sequences are in blue and mismatched bases are in red.

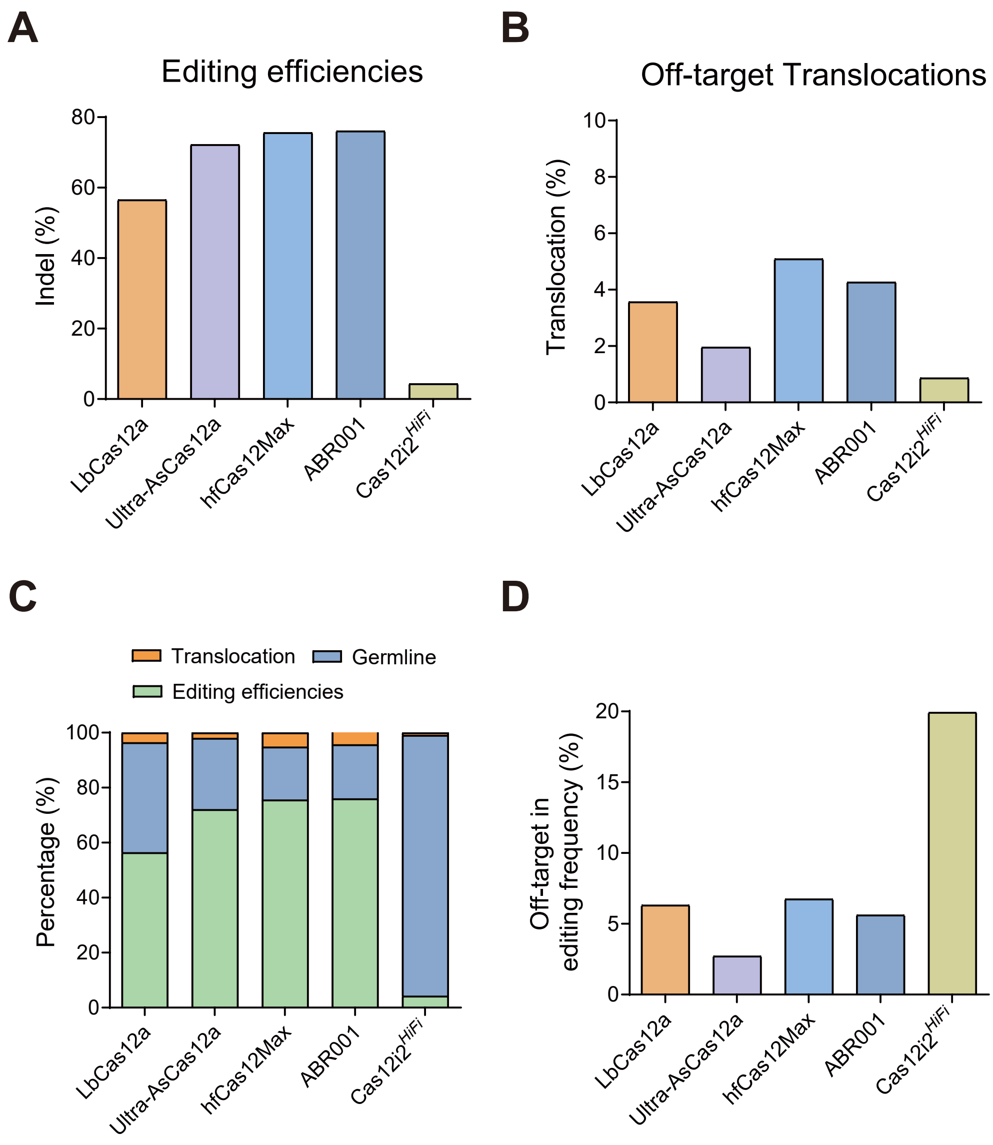

Supplementary Figure 9. Characteristics of off-target for hfCas12Max, LbCas12a, Ultra AsCas12a, ABR001 and Cas12i^{HiFi} at TTR locus detected by PEM-seq. A-C, Percentage for editing efficiencies, translocations and germline of LbCas12a, Ultra-AsCas12a, ABR001, Cas12i^{HiFi} and hfCas12Max PEM-seq libraries at TTR.2 locus in HEK293 cells. Germline represents uncut or perfect rejoining. **D,** Percentage of off-target traslocation in editing efficiencies events.

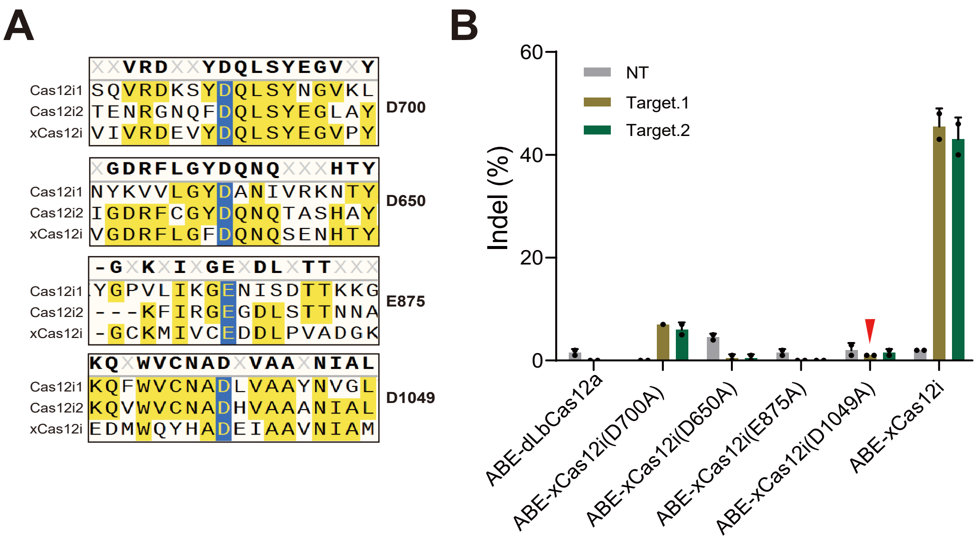

Supplementary Figure 10. Conserved cleavage sites of Cas12i. A, Sequence alignment of xCas12i, Cas12i1 and Cas12i2 shows that D650, D700, E875 and D1049 are conserved cleavage sites at RuvC domain. **B**, Introducing point mutations of D650A, E875A, and D1049A result in abolished activity of xCas12i.

A

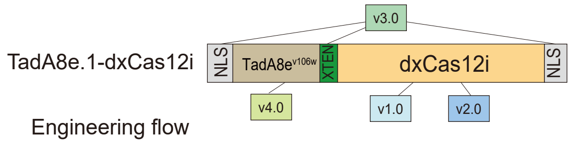

v1.0		v2.0		v3.0				v4.0			
				**v2.2**	N'	Linker	C'	**v2.2**	N'	Linker	TadA8e
v1.1	E161R	v2.1	N243R/E161R	v3.1	BpNLS	XTEN	3xSV40 NLS	v4.1	BpNLS	XTEN	W106V
**v1.2**	**N243R**	**v2.2**	**N243R/E336R**	v3.2	BpNLS	XTEN	BpNLS-cMycNLS	v4.2	cMyc-NLS	XTEN	W106V
v1.3	E336R	v2.3	N243R/D474R	v3.3	BpNLS	XTEN	CHRONO NLS	**v4.3**	**BpNLS**	**BpNLS**	**W106V**
v1.4	D474R	v2.4	N243R/Q862R	v3.4	BpNLS	XTEN	SGGS-NLS	v4.4	cMyc-NLS	BpNLS	W106V
v1.5	S245R			v3.5	BpNLS	XTEN	vBpNLS				
v1.6	N269R			v3.6	BpNLS	XTEN	BpNLS				
v1.7	S155R			v3.7	BpNLS	BpNLS	NLS				
v1.8	S268R			v3.8	cMyc-NLS	BpNLS	NLS				
v1.9	A276R										
v1.10	D287R										
v1.11	D381R										

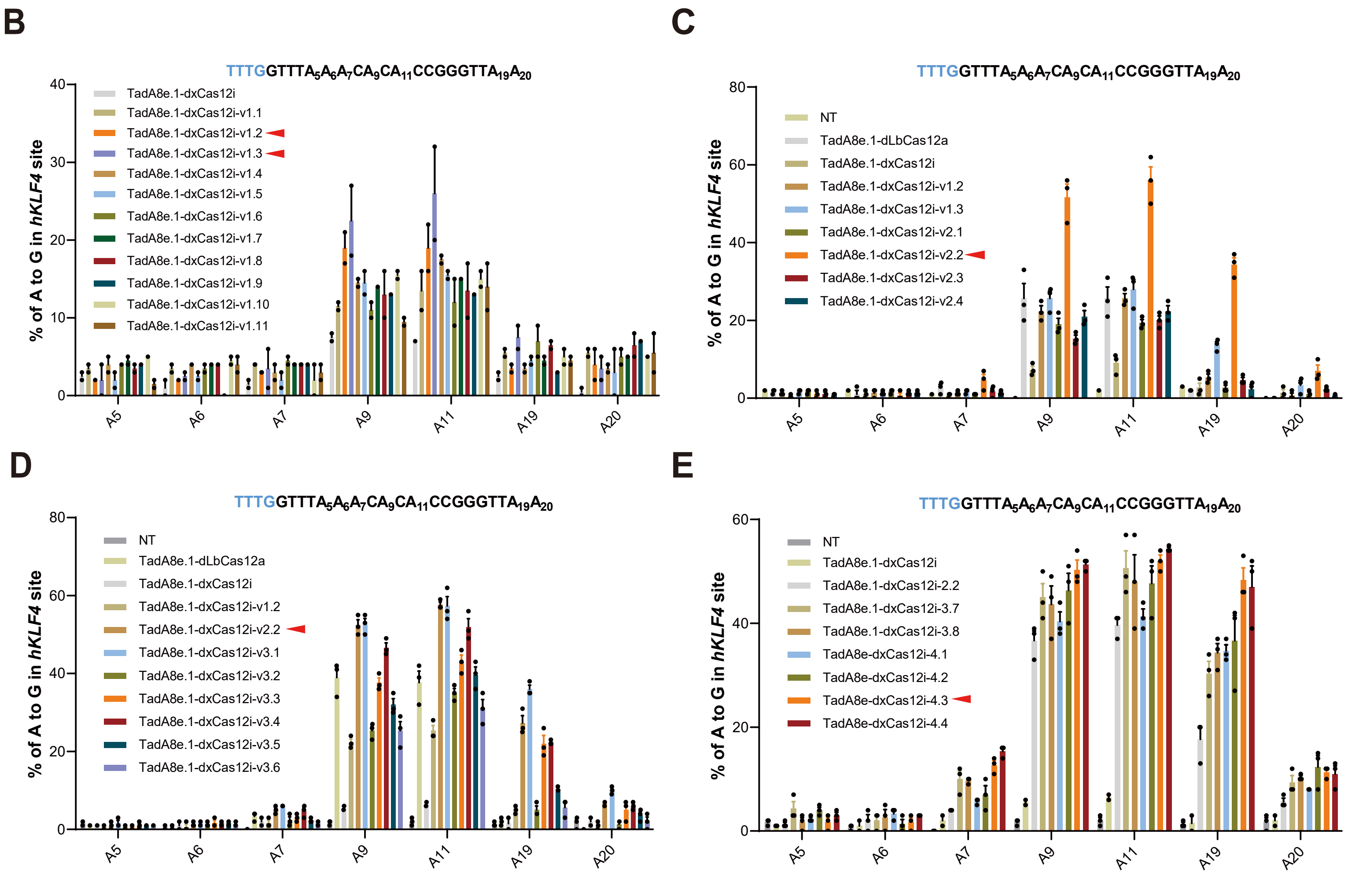

Supplementary Figure 11. Engineering for high-efficiency dxCas12i-ABE. A, Engineering schematic of TadA8e.1-dxCas12i. Four parts for engineering are indicated. **B**, TadA8e.1-dxCas12i-v1.2 and v1.3 exhibits significantly increased A-to-G editing activity among various variants at KLKF4 site of genome. **C**, Increased A-to-G editing activity of TadA8e-dxCas12i-v2.2 by combining v1.2 and v1.3. **D**, Unchanged or even decreased editing activity from various dCas12-ABEs carrying different NLS at N-terminal. **E**, Increased A-to-G editing activity of TadA8e-dxCas12i-v4.3 by combining v2.2, changed-NLS linker and high-activity Tade8e.

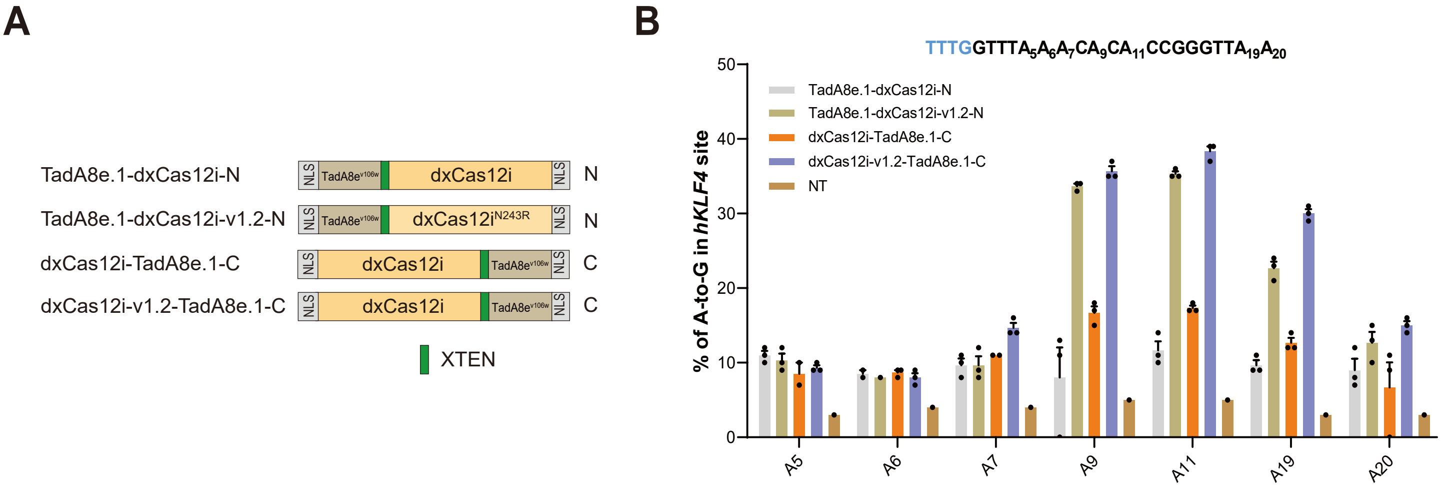

Supplementary Figure 12. Other strategies for high-efficiency dxCas12i-ABE. A, Schematics of different versions of dxCas12i adenine base editors. **B**, dxCas12i-ABE-N by TadA at the C-terminus of dCas12 slightly increased editing activity.

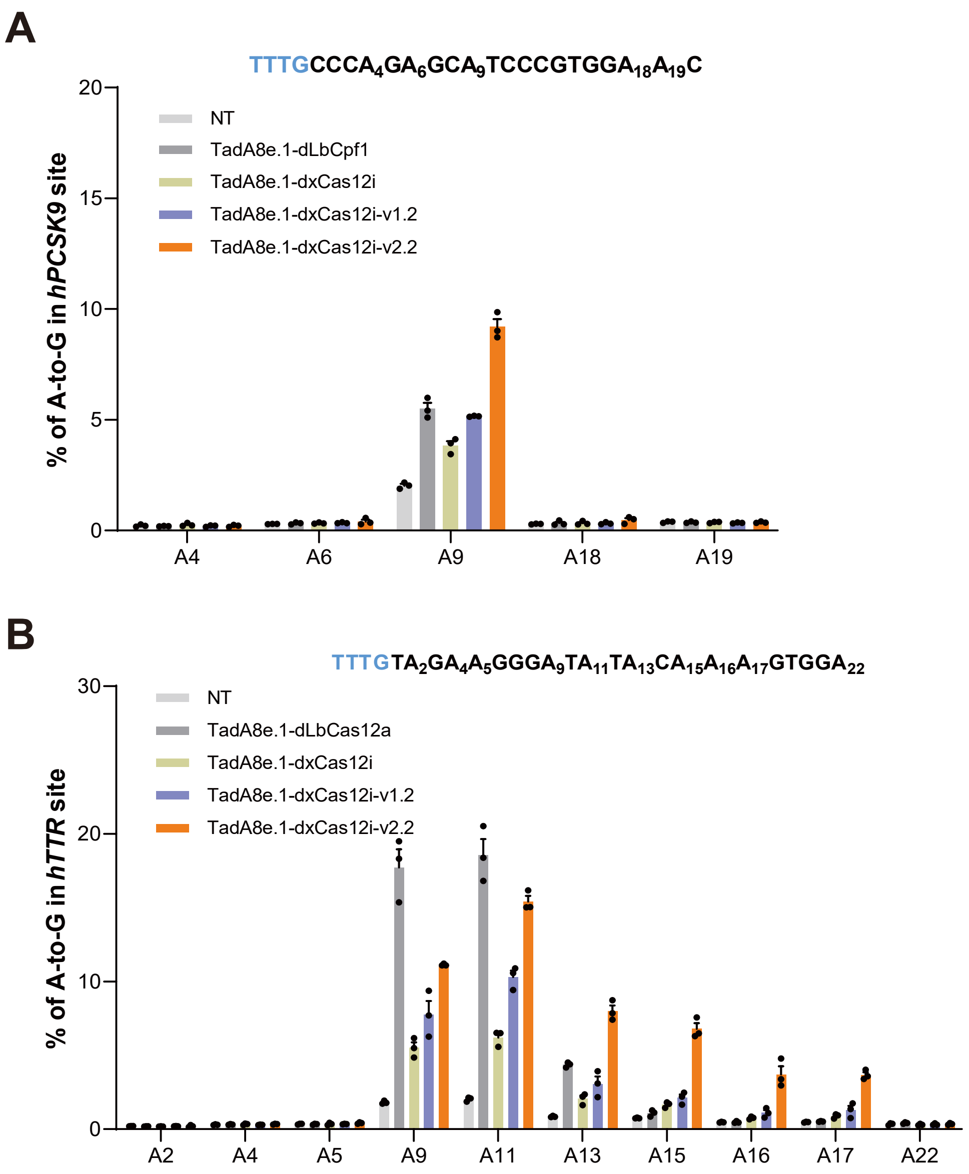

Supplementary Figure 13. Comparison of editing frequencies induced by various dCas12-ABEs at different genomic target sites. A-B, Comparison of A-to-G editing frequencies induced by indicated TadA8e.1-dxCas12i-v1.2, v2.2, and TadA8e.1-dLbCas12a at PCSK9 and TTR genomic locus.

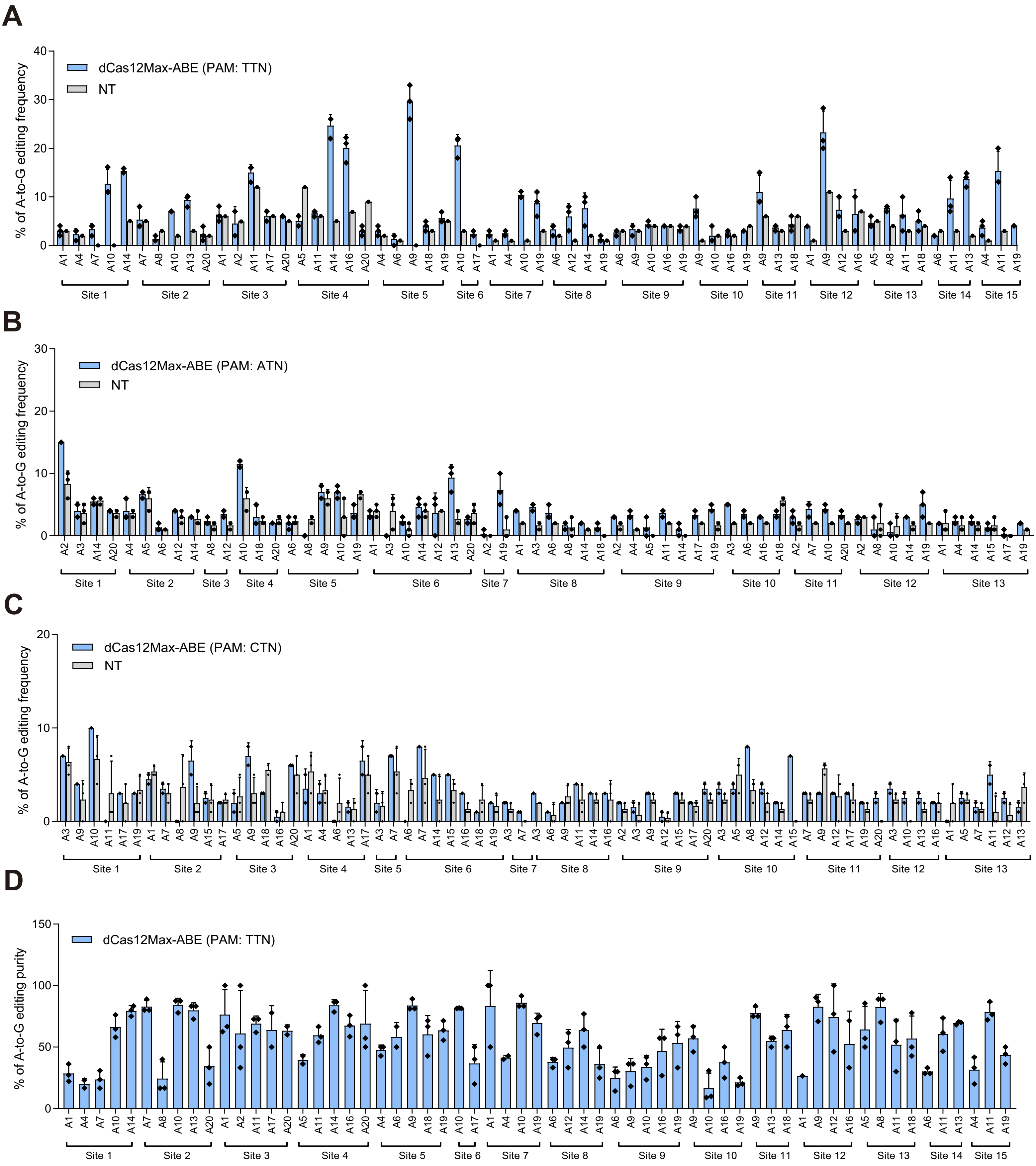

Supplementary Figure 14. Characterization of dxCas12i-ABE in HEK293T cells. A-C, dCas12-Max-ABE base editing frequency of each target sites with TTN (A), ATN (B), and CTN (C) PAM. **D**, dCas12Max-ABE base editing product purity of each target sites with TTN PAM of **A**. Target sites are indicated, with sequences of each target protospacer and PAM listed in Supplementary Table 4.

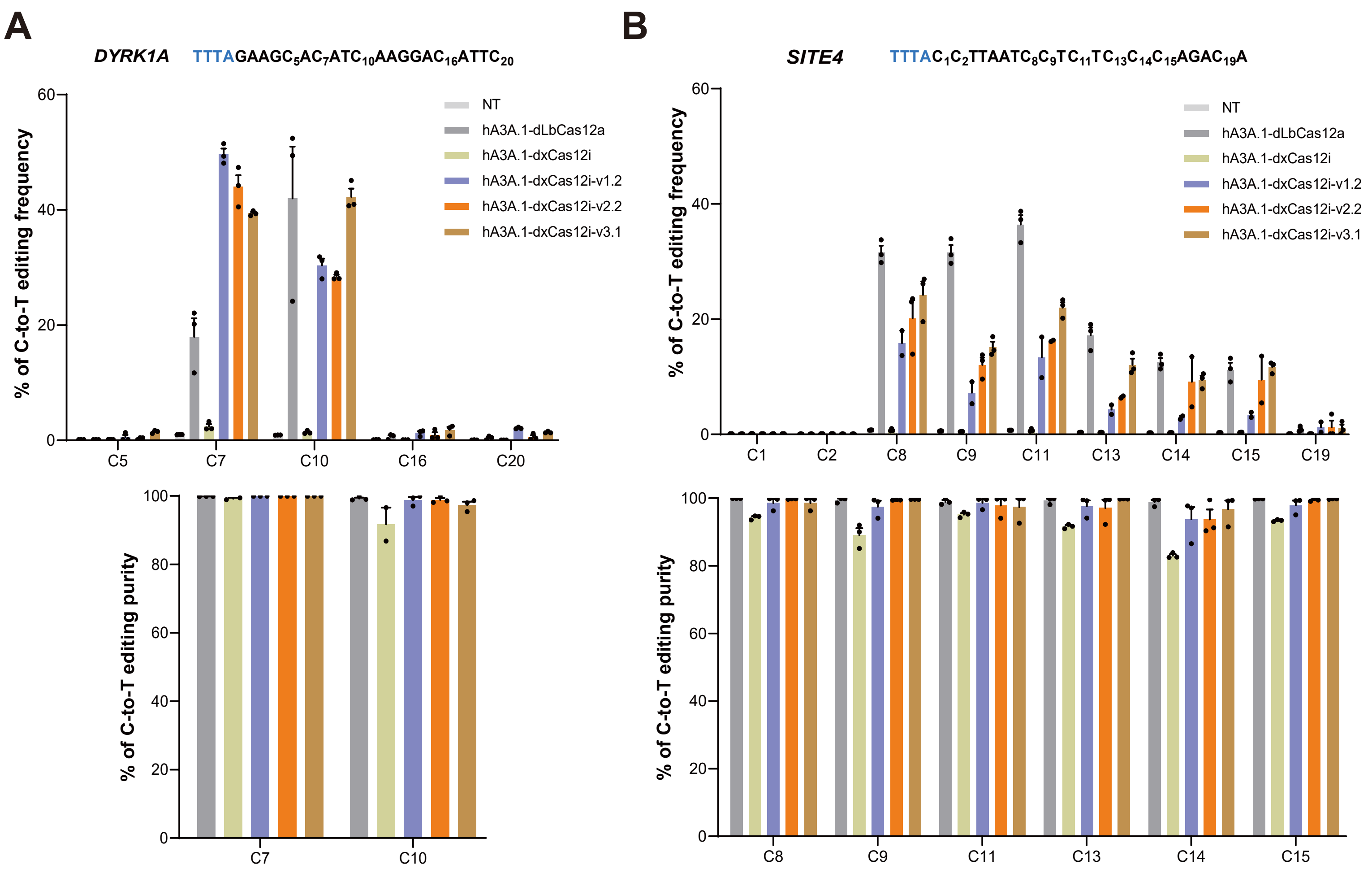

Supplementary Figure 15. Comparison of editing frequencies induced by various dCas12-CBEs at different genomic target sites. A-B, Comparison of C-to-T editing frequencies and product purity induced by indicated hA3A.1-dxCas12i, v1.2, v2.2, and hA3A.1-dCas12a at DYRK1A and SITE4 genomic locus. hA3A.1 represents human APOBEC3A^{W104A}.

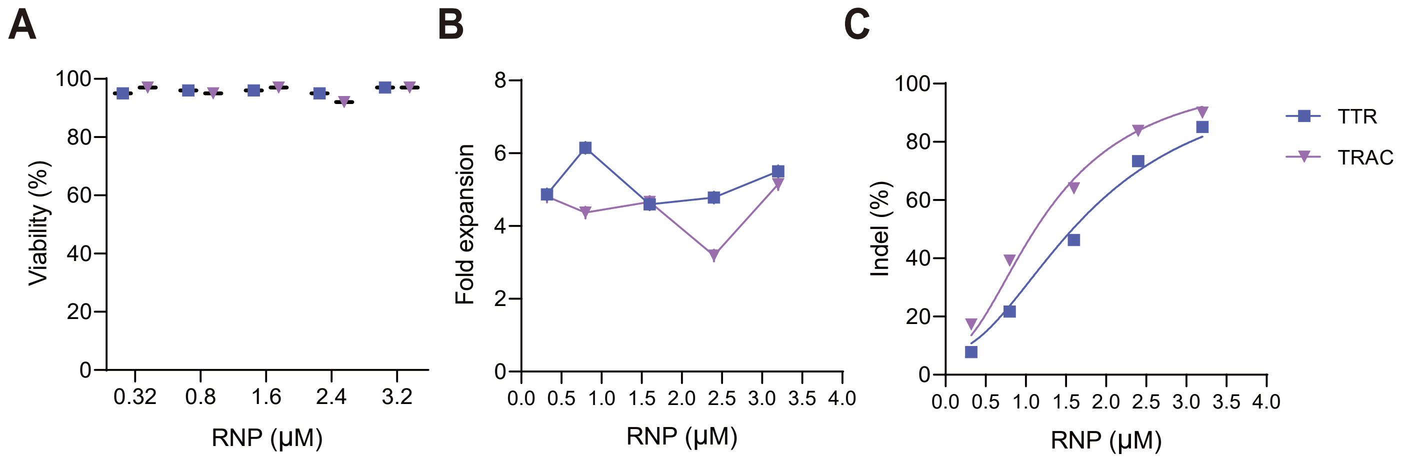

Supplementary Figure 16. hfCas12Max mediates high editing efficiency in HEK293 cells. A-C, Unchanged viability and proliferation and increasing indel activity of HEK293 cells following delivery of hfCas12Max RNPs with targeted TTR or TRAC crRNA at increasing concentration (n=1).

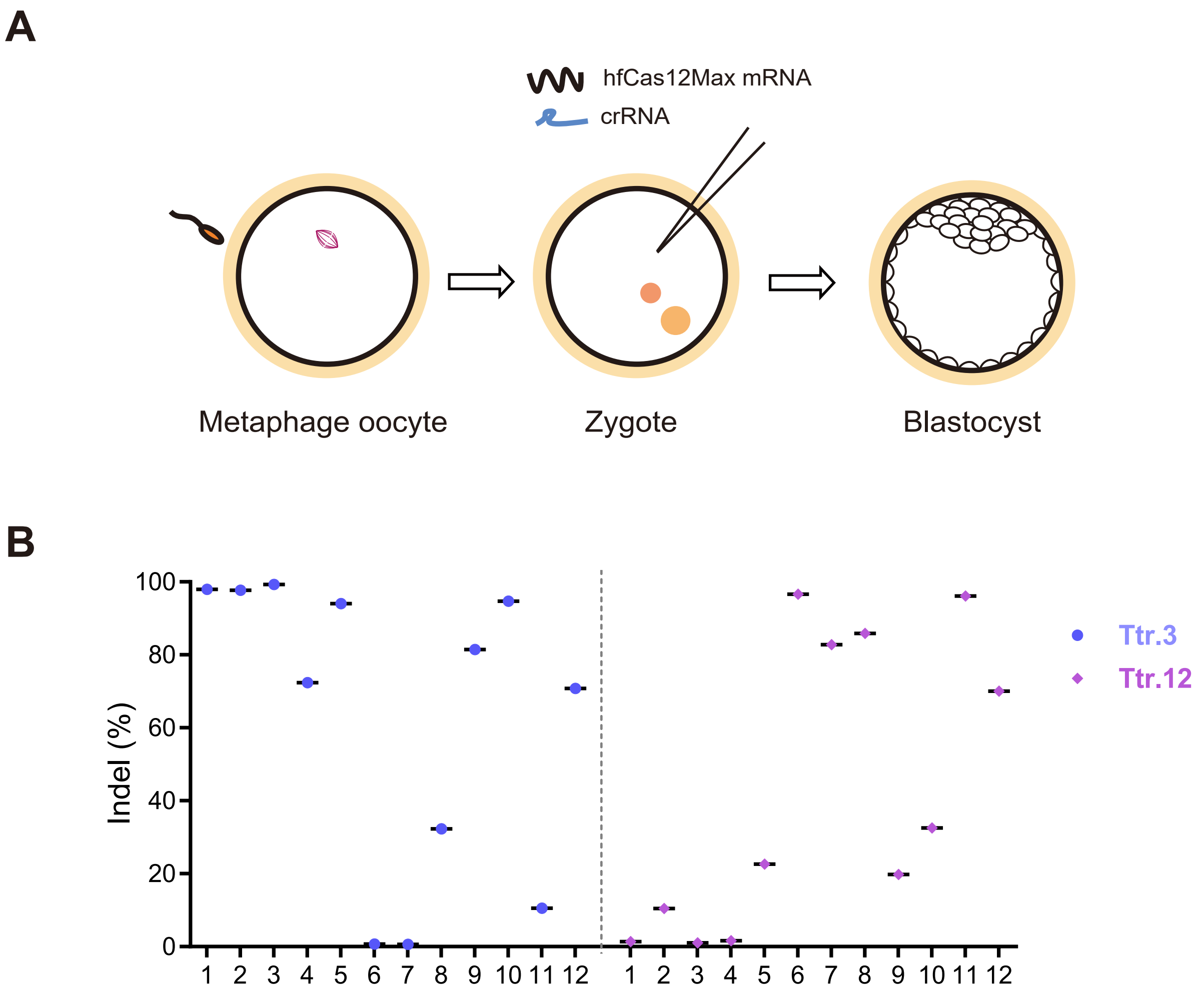

Supplementary Figure 17. hfCas12Max mediates high editing efficiency in mouse blastocyst. A, Schematics of hfCas12Max gene editing in mouse blastocyst. hfCas12Max mRNA and targeted Ttr crRNA were injected into mouse zygotes, and the injected zygotes were cultured into blastocyst stage for genotyping analysis by targeted deep sequencing. **B**, Indel rates of hfCas12Max targeted Ttr.3 and Ttr.12 in mouse blastocyst (n=12).
